# Improving the hERG model fitting using a deep learning-based method

**DOI:** 10.3389/fphys.2023.1111967

**Published:** 2023-02-06

**Authors:** Jaekyung Song, Yu Jin Kim, Chae Hun Leem

**Affiliations:** ^1^ Department of Physiology, Asan Medical Center, Seoul, South Korea; ^2^ Department of Physiology, University of Ulsan College of Medicine, Seoul, South Korea

**Keywords:** parameter inference, electrophysiology, hERG, deep learning, cardiotoxicity

## Abstract

The hERG channel is one of the essential ion channels composing the cardiac action potential and the toxicity assay for new drug. Recently, the comprehensive *in vitro* proarrhythmia assay (CiPA) was adopted for cardiac toxicity evaluation. One of the hurdles for this protocol is identifying the kinetic effect of the new drug on the hERG channel. This procedure included the model-based parameter identification from the experiments. There are many mathematical methods to infer the parameters; however, there are two main difficulties in fitting parameters. The first is that, depending on the data and model, parametric inference can be highly time-consuming. The second is that the fitting can fail due to local minima problems. The simplest and most effective way to solve these issues is to provide an appropriate initial value. In this study, we propose a deep learning-based method for improving model fitting by providing appropriate initial values, even the right answer. We generated the dataset by changing the model parameters and trained our deep learning-based model. To improve the accuracy, we used the spectrogram with time, frequency, and amplitude. We obtained the experimental dataset from https://github.com/CardiacModelling/hERGRapidCharacterisation. Then, we trained the deep-learning model using the data generated with the hERG model and tested the validity of the deep-learning model with the experimental data. We successfully identified the initial value, significantly improved the fitting speed, and avoided fitting failure. This method is useful when the model is fixed and reflects the real data, and it can be applied to any *in silico* model for various purposes, such as new drug development, toxicity identification, environmental effect, etc. This method will significantly reduce the time and effort to analyze the data.

## 1 Introduction

It is a well-known fact that it is crucial to evaluate the effects of pharmaceuticals on heart rhythm because an unstable heart rhythm causes significant problems, including death. Additionally, cardiotoxicity has resulted in the withdrawal of some previously marketed drugs and restrictions on some clinically useful drugs (Lasser et al., 2002). Therefore, there have been many discussions on the mechanisms, prevention methods, and management of such toxicity by drugs (Kelleni and Abdelbasset, 2018). In particular, screening for the *human Ether-a*`*-go-go-Related Gene* (hERG) is critical. The hERG is a gene that forms part of the rapid delayed rectifier potassium current of the heart, *I*_Kr_, and plays an important role in causing repolarization of the cardiac action potential. Many drugs that cause cardiotoxicity are known to block the hERG channel. Blockade by drugs leads to a decrease in *I*_Kr_, which can prolong ventricular action potential (Jurkiewicz and Sanguinetti, 1993). This is also associated with an increase in the QT interval (QT) in the electrocardiogram (ECG) (Sanguinetti and Tristani-Firouzi, 2006), which is likely related to Torsade de Pointes (Malik and Camm, 2001). Therefore, in 2005, the International Council for Harmonization included the following in its guidelines for non-clinical evaluation: “Preclinical Evaluation of the Possibility of Delayed Ventricular Repolarization (QT-Interval Prolongation) by Human Medicines (S7B)” (Food and Drug Administration, 2005; Friedrichs et al., 2005).

Advances in mathematical modeling and computational simulations of ion channels have made cell reactions and electrophysiological phenomena understandable and predictable, meaning they can help predict drug-induced changes. The mathematical modeling of hERG has also been continuously developed by (Zeng et al., 1995), (Beattie et al., 2018), and (ten Tusscher et al., 2004). These mathematical models are completed by fitting them to experimental data and finding the parameters. The parameters are important since they provide physiological and biophysical significance (Pathmanathan et al., 2015). However, the fitting process is by no means easy. To obtain more accurate model parameters, many mathematical and statistical methods, such as the least-squares optimization (Grisetti et al., 2020), the gradient descent (Ruder, 2016), and the Covariance Matrix Adaptation Evolution Strategy (CMA-ES) (Hansen, 2006; Khan, 2018) have been proposed and studied. Furthermore, the development of parallel computing technology and hardware has significantly aided in problem-solving by further improving the performance of these methods (Khan, 2018). However, neither method is easy to completely avoid the local minima problem, and it is time-consuming depending on the data and model. Mathematically, the best way to solve these problems is to suggest initial values close to the true values. Initial values are usually sampled from a particular distribution associated with the characteristic of the problem or given based on past experience, but none are perfect solutions.

Deep learning-based artificial intelligence (AI) has recently made tremendous progress. Great achievements have been made not only in regression and classification problems but also in the creative field. For example, in the field of vision and image processing, convolutional neural network (CNN)-based models (O’Shea and Nash, 2015), such as ResNet (He et al., 2016), EfficientNet (Tan and Le, 2019; 2021), and RegNet (Radosavovic et al., 2020), have shown better performance than humans, and in the field of time-series like natural language processing, recurrent neural network (Sherstinsky, 2020), long short-term memory (LSTM) (Hochreiter and Schmidhuber, 1997; Sherstinsky, 2020), and transformer-based models (Vaswani et al., 2017; Lin et al., 2022) are showing remarkable results.

Medicine and biotechnology also have numerous images and time-series data. Therefore, various problems can be solved through deep learning-based AI, and several studies on deep learning-based analysis are already being conducted. (Alhusseini et al., 2020; Sevakula et al., 2020; Aghasafari et al., 2021; Jeong and Lim, 2021; Rogers et al., 2021).

In this paper, we introduce a method to predict the approximate value of the hERG ion channel model parameters using a neural network and improve the fitting operation using the predicted parameters. We confirmed the performance of improved parameter fitting using the experimental data released by https://github.com/CardiacModelling/hERGRapidCharacterisation in (Lei et al., 2019).

## 2 Methods

**FIGURE 1:**
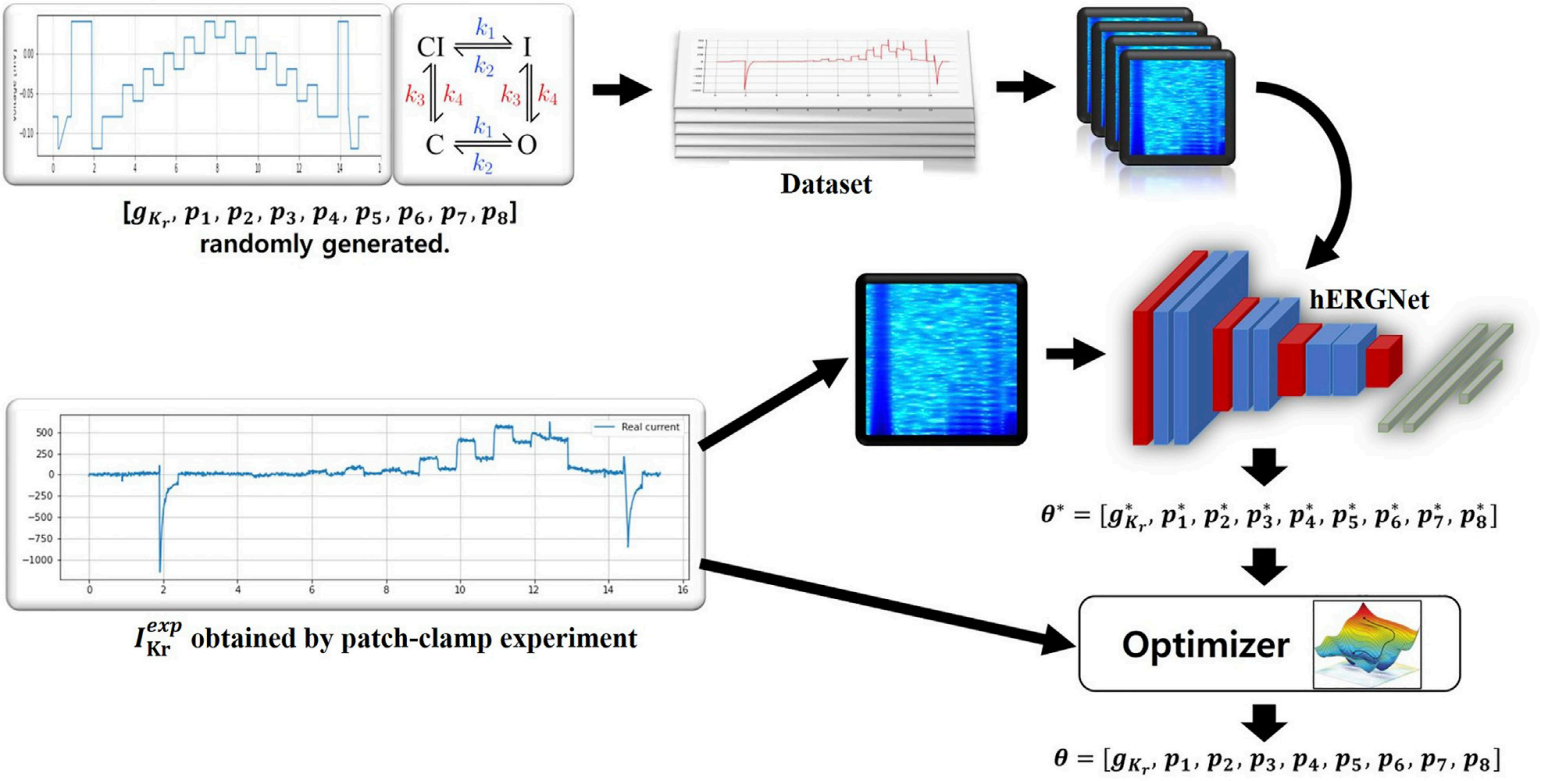
An overview of our method. A total of 500,000 were generated to train hERGNet, and each data consisted of ( $I_{Kr}^{sim}$ , θ ). The trained hERGNet predicts $\theta^{*}$ through $I_{Kr}^{exp}$ obtained by a patch-clamp experiment. Parameter inference is performed using $\theta^{*}$ as the initial value.

Our method is as follows. First, the simulation generates $I_{Kr}$ current data. The generated current data was then converted into a spectrogram. Next, our parametric prediction network was trained from the generated simulation data. The experimental data were used only for the validation and testing of the network. Using the trained prediction network, nine parameters were predicted based on the current generated by the patch-clamp experiment. Figure 1 depicts the overall overview.

### 2.1 hERG model

**FIGURE 2:**
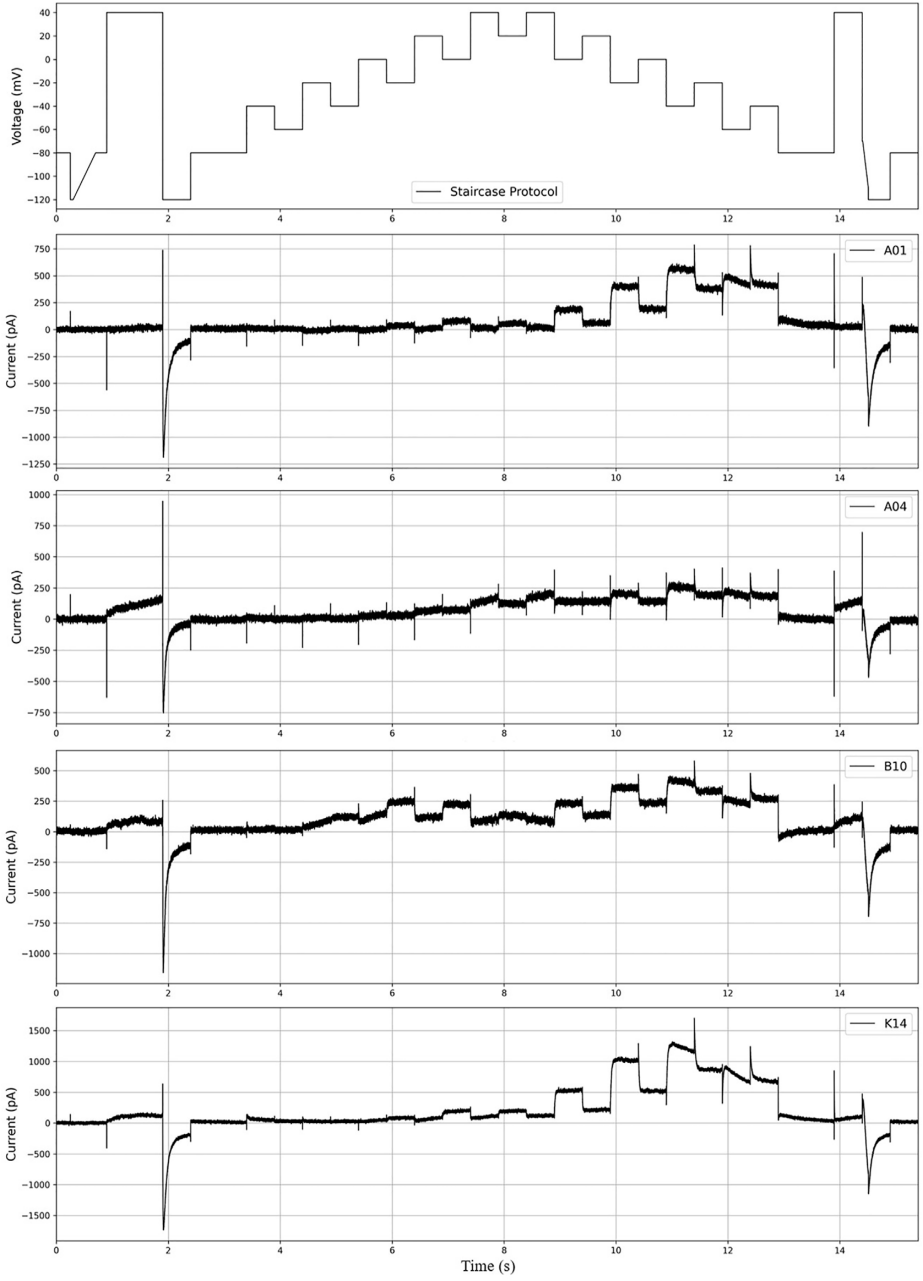
The figure above shows the “staircase protocol” introduced in (Lei et al., 2019) and the $I_{Kr}$ currents for cells “A01,” “A06,” “B10,” and “K14.”

In this study, we used the experimental data published in (Lei et al., 2019, https://github.com/CardiacModelling/hERGRapidCharacterisation). Therefore, the hERG model and basic settings we use are the same as those of (Lei et al., 2019). For ease of training and evaluation of our deep learning model, we excluded 11 of the 211 cells, which seemed to have a large difference between the experimental results and the results produced by the hERG model. The currents were recorded for the “staircase protocol” (Figure 2). As shown in the top image in Figure 2, each step is 500 ms, long enough to see the characteristics of $I_{Kr}$ . Thus, it is possible to observe the dynamics at different voltage values. Lei et al. showed that their protocol provided enough information to infer true parameters through a synthetic data study (Lei et al., 2019).

This hERG model with Hodgkin and Huxley style structure used in this experiment is Beattie’s model (Beattie et al., 2018) slightly improved by ten Tusscher et al. (ten Tusscher et al., 2004), and *I*_Kr_ is the same as Eq. (1).

$$I_{Kr}=g_{Kr}ar\left(V-E_{K}\right)$$ (1)

where $g_{Kr}$ is the maximum conductance, and a and r denote a Hodgkin and Huxley activation gate and an inactivation gate, respectively. V is the transmembrane voltage. $E_{K}$ is called Nernst potential or the reversal potential and obtained by Eq. (2).

$$E_{K}=\frac{RT}{zF}\ln\left(\frac{{\left[K^{+}\right]}_{o}}{{\left[K^{+}\right]}_{i}}\right)$$ (2)

where

$$R\;\left(ideal\;gas\;constant\right):8.314472\;\left[\frac{J}{K∙mol}\right]$$

$$T\;\left(absolute\;temperature\right):298.15\;\left[K\right]$$

$$F\;\left({Faraday}^{\prime }s\;constant\right):96485.3415\;\left[\frac{C}{mol}\right]$$

$$z\;\left(valency\;of\;the\;ions\right):+1\;for\;K^{+}$$

$${\left[K^{+}\right]}_{o}\;\left(extracellular\;concentration\right):4\;\left[mM\right]$$

$${\left[K^{+}\right]}_{i}\;\;\left(intracellular\;concentration\right):110\;\left[mM\right]$$

**FIGURE 3:**
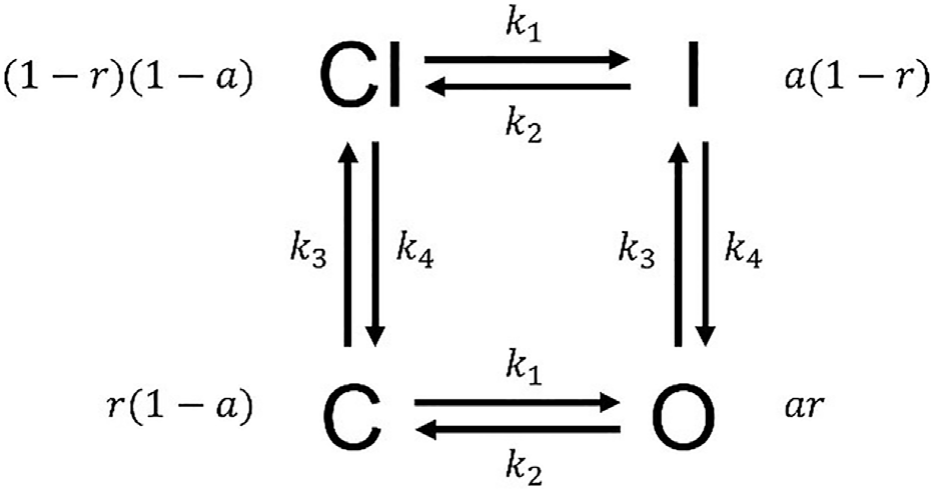
The Hodgkin–Huxley model structure. The probabilities for states CI, I, O, and C are $\left(1-r\right)\left(1-a\right)$ , $a\left(1-r\right)$ , ar and $r\left(1-a\right)$ .

The model has nine parameters $\theta =\left\{g_{Kr},p_{1},p_{2},p_{3},p_{4},p_{5},p_{6},p_{7},p_{8}\right\}$ , and Figure 3 shows its structure, where

$$\begin{array}{cc} \frac{da}{dt}=\frac{a_{\infty }-a}{\tau_{a}} & ,\frac{dr}{dt}=\frac{r_{\infty }-r}{\tau_{r}}\\ a_{\infty }=\frac{k_{1}}{k_{1}+k_{2}} & ,r_{\infty }=\frac{k_{4}}{k_{3}+k_{4}}\\ \tau_{a}=\frac{1}{k_{1}+k_{2}} & ,\tau_{r}=\frac{1}{k_{3}+k_{4}} \end{array}$$

$$\begin{array}{c} k_{1}=p_{1}\exp\left(p_{2}V\right)\\ k_{2}=p_{3}\exp\left(-p_{4}V\right)\\ k_{3}=p_{5}\exp\left(p_{6}V\right)\\ k_{4}=p_{7}\exp\left(-p_{8}V\right) \end{array}$$

where $k_{1}$ is an activation rate, $k_{2}$ is a deactivation rate, $k_{3}$ is an inactivation rate, and $k_{4}$ is a recovery rate.

### 2.2 Dataset generation

A lot of data is required to train. The best condition for good performance is the presence of a large amount of experimental data. However, there are only 200, and it is very insufficient for learning and testing with them. Even if a large amount of data exists, fitting work for labeling requires a lot of time and resources, which is contrary to the purpose of our study. So, we generated a large amount of data using simulations of the hERG channel to compensate for the lack of data. The dataset consists of *I*_Kr_ as input data and nine parameters θ of the hERG model as the target data. Five hundred thousand data generated by simulation were used for training. For 200 experimental data, after sorting by name, odd-numbered data were configured as validation dataset and even-numbered data were configured as test dataset.

**FIGURE 4:**
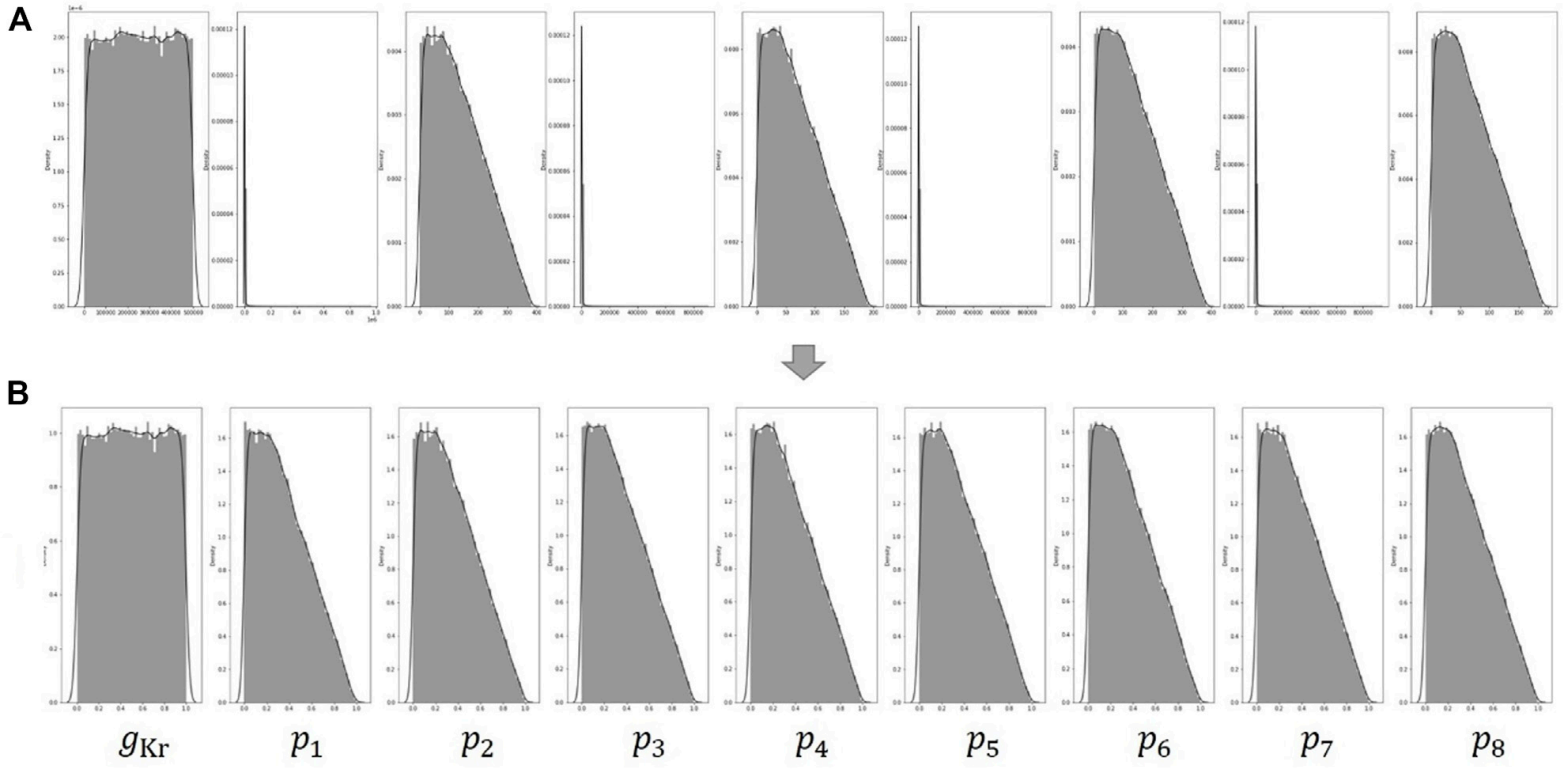
**(A)** Distribution of the nine parameters generated by the two conditions of Eqs 3, 4. The distributions of $p_{1}$ ; $p_{3}$ ; $p_{5}$ ; $p_{7}$ are very clustered in a specific range. **(B)** Data distributions after applying log scale to $p_{1}$ , $p_{3}$ , $p_{5}$ and $p_{7}$ .

First, parameters for the hERG channel were generated under the following two conditions. The first condition is that each parameter follows a uniform distribution within a specific range, as in Eq. (3).

$$g_{Kr}∼U\left(g_{\min},g_{\max}\right)$$

$$p_{1},p_{2},p_{3},p_{4}\;∼U\left(a,b\right)$$ (3)

$$p_{5},p_{6},p_{7},p_{8}\;∼U\left(c,d\right)$$

where $U\left(∙\right)$ represents a uniform distribution. In this study, for the conductance $g_{Kr}$ , $g_{\min}=100$ [pA/V] and $g_{\min}=500000$ [pA/V]. For $p_{1},p_{3},p_{5},\mathrm{and}p_{7}$ , a and b are 0.0001 [s^−1^] and 10^6^ [s^−1^], respectively. For $p_{2},p_{4},p_{6},\mathrm{and}p_{8}$ , c and d are 0.0001 [V^−1^] and 400 [V^−1^], respectively. The second condition is that each parameter must satisfy the inequalities of Eq. (4):

$$0.0167<p_{1}\exp\left(p_{2}*V_{\max}\right)<10^{6}$$

$$0.0167<p_{3}\exp\left(-p_{4}*V_{\min}\right)<10^{6}$$ (4)

$$0.0167<p_{5}\exp\left(p_{6}*V_{\max}\right)<10^{6}$$

$$0.0167<p_{7}\exp\left(-p_{8}*V_{\min}\right)<10^{6}$$

where $V_{\min}=-0.12$ and $V_{\max}=0.06$ . The lower and upper bounds of the above conditions were determined by the constraints of physical and physiological phenomena (Beattie et al., 2018). Figure 4A shows the distribution of each parameter for 100,000 data generated by the above conditions.

Second, we used simulations to generate the current $I_{Kr}$ corresponding to the parameters sampled above. Myokit (Clerx et al., 2016) with CVODE solver (Hindmarsh et al., 2005) was used for the simulation. The tolerance settings for CVODE were *abs_tol* = 10^–8^ and *rel_tol* = 10^–10^, as in the condition in (Lei et al., 2019). The length of the experimental data was 15.4 s with a sampling rate of 5 kHz. In this study, we reduced the data number to 1/50 with sampling one point every 100 because there seemed to be no problem reflecting the trend. Because we only used the data generated by simulations for training, the results of this study depend on the similarity between the simulation data and experimental data. Therefore, noises, α , were added to the simulation data. The noises were extracted from a normal distribution $\alpha ∼N\left(0,\sigma^{2}\right)$ . σ is 10.84, which was measured at the steady-state current in the experimental data.

$$I_{Kr}^{\exp}\approx I_{Kr}^{sim}+\alpha$$ (5)

where $I_{Kr}^{exp}$ and $I_{Kr}^{sim}$ represent the experimental and simulated current, respectively.

### 2.3 Preprocessing and hERGNet

Our method involves several simple preprocessing processes on the data for learning. In Figure 4A, The distributions of $p_{1}$ , $p_{3}$ , $p_{5}$ and $p_{7}$ are very clustered in a specific range. To make them as uniform as possible, the log scale was applied. The min–max normalization was then used to transform all parameter ranges between 0 and 1, as shown in Figure 4B.

**FIGURE 5:**
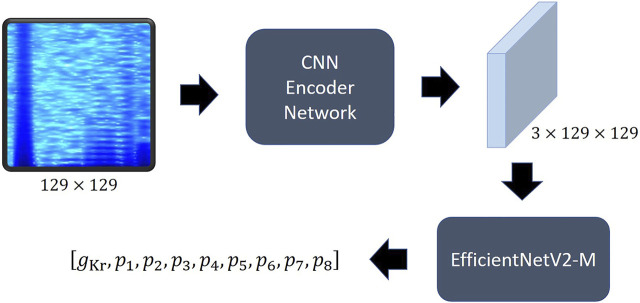
The structure of hERGNet. The spectrogram of current is converted into 2D data with three channels like the image shape through the CNN encoder, making it possible to utilize the pretrained EfficientNet.

Recurrent neural network (Sherstinsky, 2020) and LSTM (Hochreiter and Schmidhuber, 1997; Sherstinsky, 2020) series models have been widely used to analyze time-series data, such as the current data that we want to analyze, and recently, transformer-based models (Vaswani et al., 2017; Lin et al., 2022) are leading this field. Since CNN is designed for the purpose of extracting information between adjacent values of data, it obtains spatial information well in the local domain (Krizhevsky et al., 2017). Transformer calculates the relationship between all elements of input data through attention, so it understands overall features better than CNN, but is weaker than CNN in extracting local information, and requires a very large size dataset for this purpose (Dosovitskiy et al., 2020). We thought our goal was closer to finding changes in the characteristics and patterns at specific times than predicting the current change over time. Therefore, we adopted a CNN-based model rather than a transformer-based model, and among them, an EfficientNet (Tan and Le, 2019) type model. We called our model hERGNet. Our hERGNet is very simple, as shown in Figure 5. It consists of a CNN-encoder network and a pretrained EfficientNetV2-M (Tan and Le, 2021). The CNN-encoder network extracts the features of the spectrogram and increases the number of channels to three to obtain an image-like shape, thereby making it possible to use the pretrained EfficientNetV2-M. EfficientNetV2-M is responsible for finding parameters by extracting features from encoded data.

**FIGURE 6:**
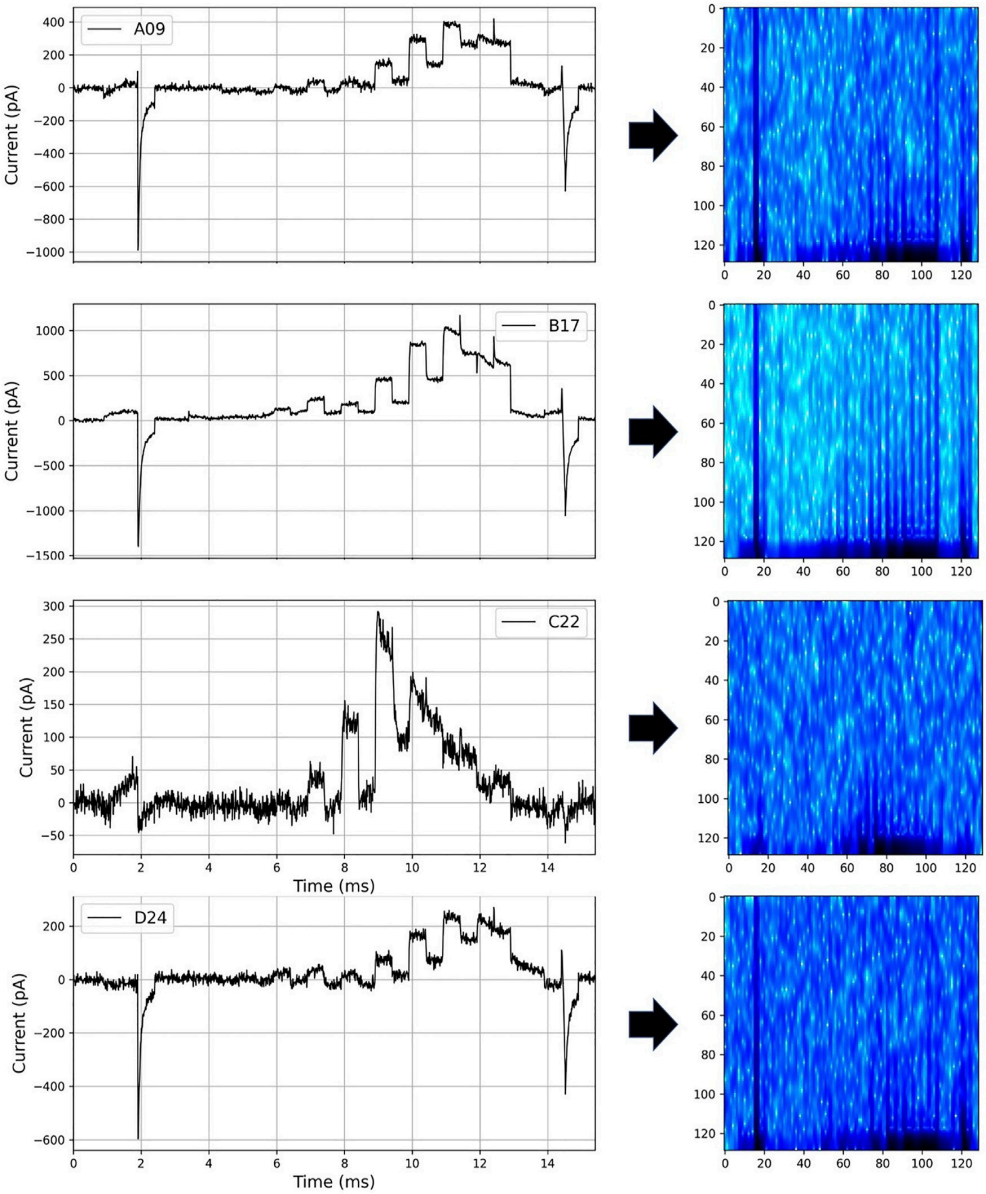
Spectrograms transformed from current data for 4 cells.

We converted the current into a spectrogram with a frequency perspective, as shown in Figure 6, so that hERGNet can better learn the characteristics of the current data. By adding frequency features to current data consisting of only time and intensity, it has the advantage of increasing information about data and transforming it into a two-dimensional form like an image, making it easier for a 2D CNN-based model to learn. In this study, the parameters “n_fft,” “hop_length,” and “win_length” of Short-Time Fourier Transform (Owens and Murphy, 1988) for spectrogram transformation were set to 256, 12, and 48, respectively.

### 2.4 Training

Mean Squared Error (MSE) was used as the loss function, so the cost function J(θ) is the same as Eq. (6)

$$\begin{array}{r} J\left(\theta \right)=\frac{1}{N}\overset{N}{\underset{i=1}{\sum }}\left(f_{\theta }\left(x_{i}\right)-y_{i}\right) \end{array}$$ (6)

where $x_{i}$ is the spectrogram of the current $I_{Kr}$ , $f_{\theta }$ is the neural network, and $y_{i}$ is the true parameters of the ion channel.

In general, it is known that the higher the resolution of the input, the higher the performance. However, the higher the resolution, the more memory is required, which is time-consuming to train due to the small batch size. Also, if you train all the generated 500,000 data from the beginning, the time will increase even more. Therefore, we first trained the hERGNet with a small resolution and a small number of data and then proceeded with transfer learning by increasing the resolution and the number of data. First, learning was performed on 300,000 spectrograms with a 97 × 97 resolution to 200 epochs. Then, we increased the resolution to 129 × 129 and performed transfer learning to 140 epochs using all 500,000 data.

### 2.5 Parameter inference

All fitting operations were the same as those in (Lei et al., 2019), and their open-source library PINTS (Clerx et al., 2019) was used. Furthermore, the CMA-ES algorithm (Hansen, 2006; Khan, 2018) was used as a global optimization algorithm to fit the model to the experimental data, and Markov Chain Monte Carlo (Jasra et al., 2007) with adaptive Metropolis (Haario et al., 2001) was used to explore the posterior probability distribution. Parameter inference was performed three times based on the initial value for each cell. The first was when the initial value was given as a parameter predicted by our hERGNet, the second was a prior value, and the last was given as a value randomly extracted from the previously described parameter distribution.

## 3 Results

**FIGURE 7:**
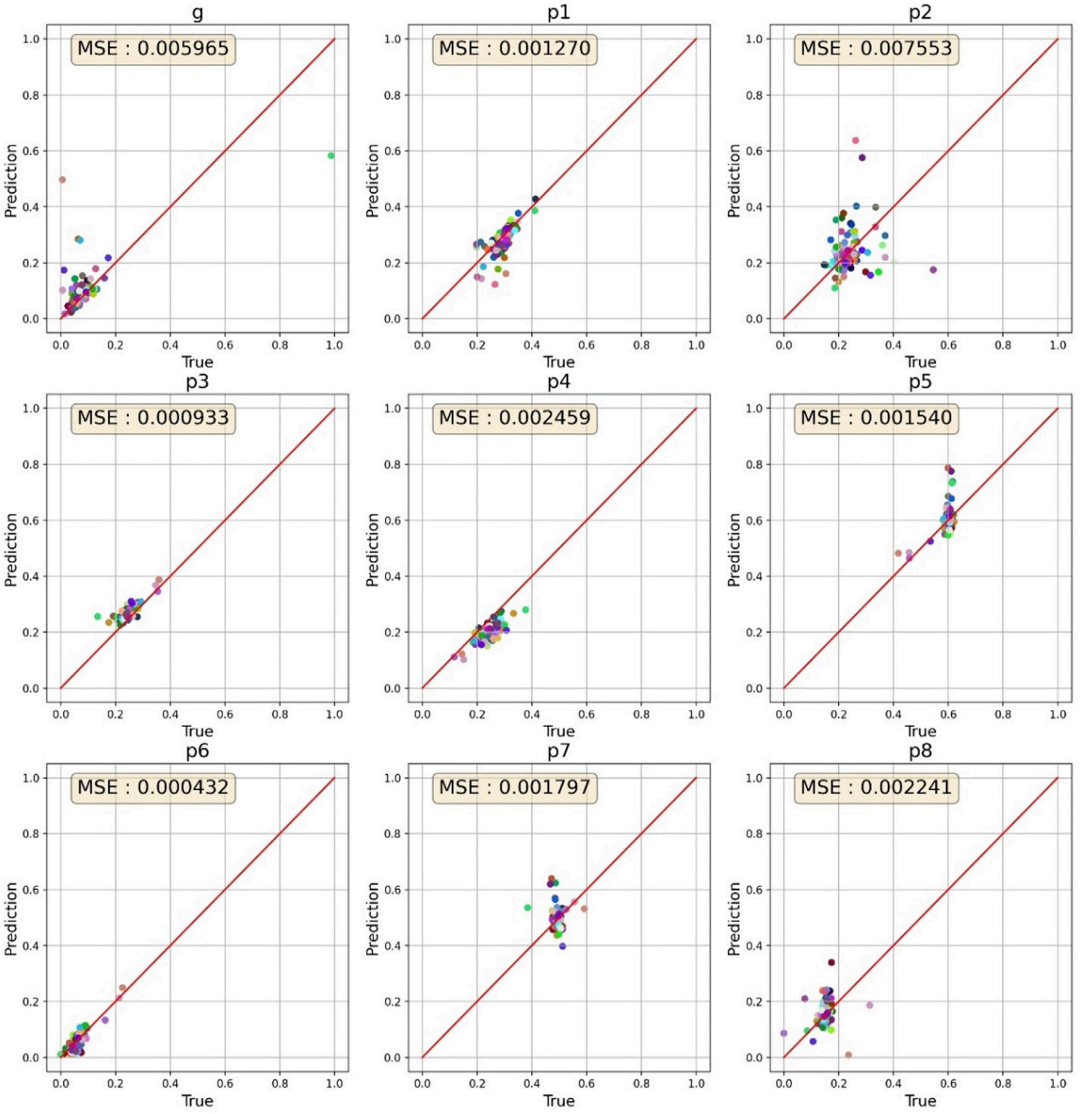
Prediction results for 100 cells. The parameters for most cells are clustered in a specific range. $p_{6}$ had the best predictions, while $p_{2}$ had the worst predictions.

When hERGNet was trained on 300,000 data with a 97 × 97 resolution until 200 epochs, the best MSE for 100 experimental data (validation data) was 0.00781. After 140 additional learnings on 500,000 data with an increased resolution to 129 × 129, we identified MSEs lowered to 0.003035. Then, we tested hERGNet on 100 experimental data not used for learning. The MSE was recorded as 0.002688, which is better than the results for the validation dataset. Figure 7 shows the prediction results for 100 experimental data. The fitting test was performed on 50 experimental data out of the 100 test data.

**FIGURE 8:**
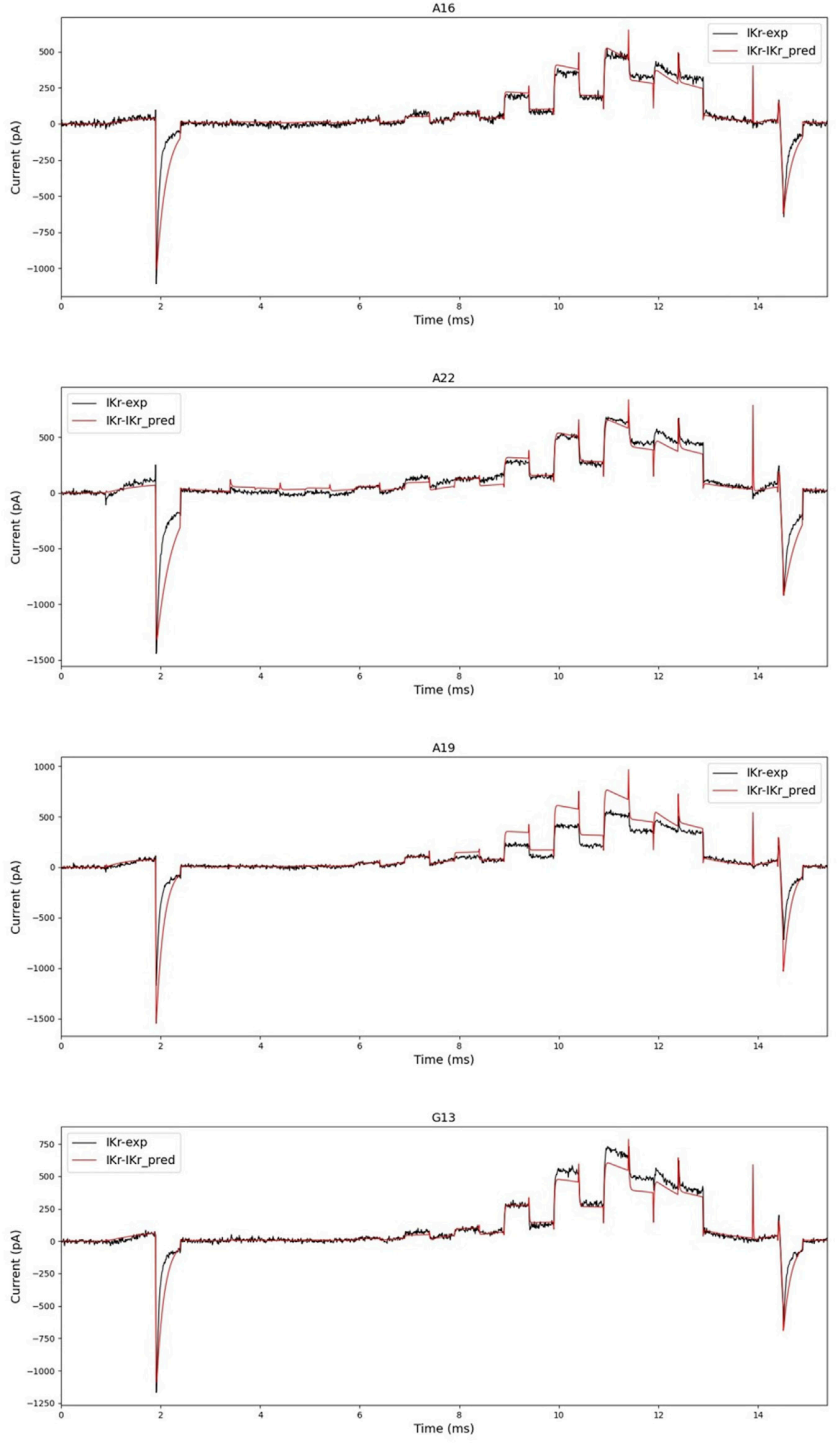
Black is experimental data, and red is simulated current data with parameters predicted by hERGNet. There are some very close predictions, such as A16 and A22, and results showing differences, such as A19 and G13. However, the flow and shape of the current are somewhat predictable.

First, when we generated current data with the predicted parameters, we compared how different it was from the experimental data as shown in Figure 8. As shown in the two figures above in Figure 8, the prediction was accurate enough that no fitting work was required for a significant number of cells. However, as shown in the two figures below in Figure 8, the prediction was not perfect, necessitating a fitting operation.

Next, as shown in Table 1, we compared the results when the initial values were given as parameters predicted by hERGNet, prior parameters, and random parameters. The results confirmed that our method significantly improved the fitting operation. The initial value of hERGNet did not result in a single failure in the fitting operation for 50 cells. However, the prior parameter caused one local minima problem, and in the random parameter, 16 failures occurred out of 50 fittings, and 11 local minima problems occurred. We compared the fitting rates for cells that succeeded in parameter inference. As shown in Table 1, the average iteration was 341.8 in the predicted parameter, 546.4 in the prior parameter, and 601.3 in the random parameter. The average time was 396.3 s for the predicted parameters, 630.9 s for the prior parameter, and 686.0 s for the random parameters. Of the 50 cells, all but two, D17 and G13, showed faster fitting rates when using the predicted parameters.

**TABLE 1:** When the predicted value by hERGNet was used, there was a great improvement in the fitting speed.

	Predicted	Prior	Random
Average Iteration	341.8	546.4	601.3
Average Time (s)	396.3	630.9	686.0

## 4 Discussion and conclusion

Parameter inference is an important part of the toxicity evaluation of drugs because it is possible to understand and predict physiological changes in cells caused by drugs by predicting the parameters of ion channels. However, the difficulty of the fitting and the time-consuming problem make us hesitant to use *in silico*. In this study, we propose a method for improving the fitting operation for the hERG channel model by setting the parameters predicted by hERGNet as initial values. The test results showed a clear improvement in the hERG model fitting. There was no fitting failure, and the time-consuming problem was also improved. Depending on the range of parameters, training the neural network required a lot of data generation and was time-consuming. However, if experiments are conducted with the same voltage protocol for other cells in the future, our method could be very useful for inferring the parameters of ion channels.

Our method still has a lot to improve. The first is to improve the fitting method rather than simply presenting initial values. This is because stochastic methods, such as CMA-ES, may not immediately find optimal parameters due to the characteristics of the method, even if parameters close to the correct answer are presented. The second is to increase the similarity between experimental and simulation data, which is, after all, the most important factor for AI to predict parameters. We trained our hERGNet only with simulation data. If the similarity between experimental and simulation data can be increased through noise removal, etc., the predicted parameters will be closer to the correct answer.

In fact, our ultimate goal is to predict parameters that are very close to the correct answer, eliminating the need for model fitting. If this is possible, a new paradigm will be presented in drug development or drug toxicity assessment. To this end, we will first conduct a study on parameter prediction in multiple ion channels. Parametric prediction for multiple ion channels may aid in greatly reducing the amount and cost of experiments performed in the non-clinical stage.

## Data Availability

The original contributions presented in the study are included in the article, further inquiries can be directed to the corresponding author.
